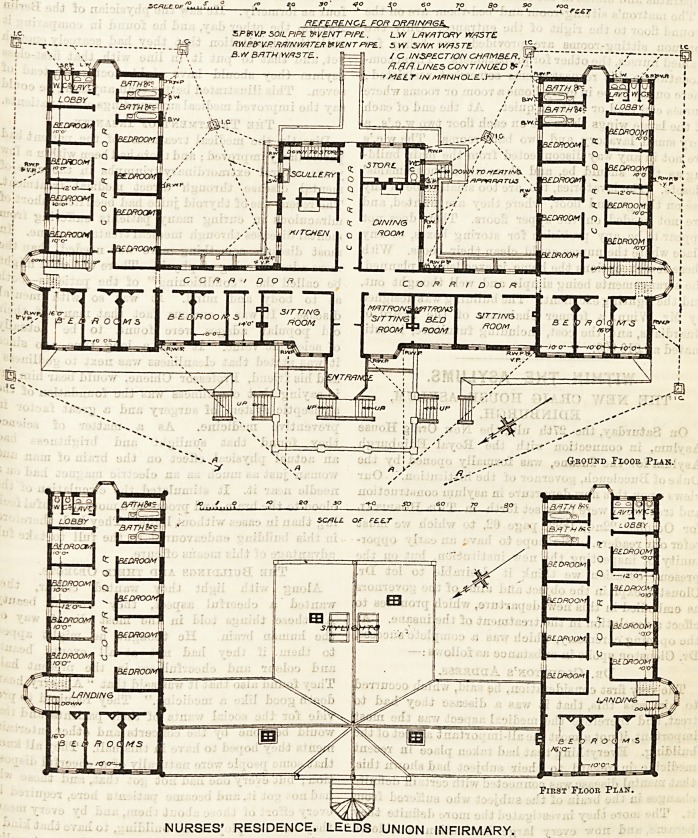# Nurses' Home at Burmantofts (Leeds Union Infirmary)

**Published:** 1894-11-03

**Authors:** 


### The Institutional Workshop.

### HOSPITAL CONSTRUCTION.

### NURSES' HOME AT BURMANTOFTS.
### (LEEDS UNION INFIRMARY).

The great improvements that have taken place of late years in the nursing of workhouse infirmaries has rendered necessary corresponding improvements in the housing of the nurses. The new home at Burmantofts in connection with the Leeds Union Infirmary, which is course of construction, is a case in point. The authorities had experienced great difficulty in obtaining and keeping an efficient nursing staff, and inquiries made from time to time showed that this was largely due to the inferior character of the accommodation provided, and that the provision of suitable quarters for nurses in other large institutions of the kind attracted nurses away from those infirmaries where no improvements had been made.

The new building which the Leeds Guardians are providing for their nurses is situated about one hundred

NURSES' RESIDENCE, LEEDS UNION INFIRMARY.

and thirty yards behind the infirmary, with a large space of open ground in front of it. The ground plan is in the form of an E, of which the two outer wings only are two storeys high, the remainder being one storey. Provision is made for forty nurses in separate rooms, averaging 10 feet by 12 feet each.

The central wing at the back contains the kitchen offices and the general dining-room, with a heating apparatus and stores in the basement.

The matron's sitting-room and bed-room are on the ground floor to the right of the entrance. Only two common sitting-rooms are provided, one being for trained nurses, the other for probationers. The accommodation in this respect seems somewhat restricted; there ought to be in every home a room or rooms where nurses can read or write in quiet. At the end of each of the large wings there are on each floor two w.c.'s, a very small lavatory, and two bath-rooms. The w.c.'s are not in any way disconnected from the main building as they should be, and there are no housemaids' closets. The lavatories, too, are too small to be of any use on the ground floor, where they are wanted, and are not needed on the upper floors. There does not appear to be any provision for storing boxes, or any place where the nurses could clean their boots. With these few exceptions the home is excellently planned, the arrangements being simple and well thought out, and the lighting excellent. The building was designed by Mr. Winn, a former chairman of the Board of Guardians, and the cost, including furniture, is estimated at £10,000.

## Figures and Tables

**Figure f1:**